# Development and validation of risk prediction equations to estimate future risk of heart failure in patients with diabetes: a prospective cohort study

**DOI:** 10.1136/bmjopen-2015-008503

**Published:** 2015-09-09

**Authors:** Julia Hippisley-Cox, Carol Coupland

**Affiliations:** Division of Primary Care, University Park, Nottingham, UK

**Keywords:** EPIDEMIOLOGY, PREVENTIVE MEDICINE, PRIMARY CARE

## Abstract

**Objective:**

To develop and externally validate risk prediction equations to estimate the 10-year risk of heart failure in patients with diabetes, aged 25–84 years.

**Design:**

Cohort study using routinely collected data from general practices in England between 1998 and 2014 contributing to the QResearch and Clinical Research Practice Datalink (CPRD) databases.

**Setting:**

We used 763 QResearch practices to develop the equations. We validated it in 254 different QResearch practices and 357 CPRD practices.

**Participants:**

437 806 patients in the derivation cohort; 137 028 in the QResearch validation cohort, and 197 905 in the CPRD validation cohort.

**Measurement:**

Incident diagnosis of heart failure recorded on the patients’ linked electronic General Practitioner (GP), mortality, or hospital record. Risk factors included age, body mass index (BMI), systolic blood pressure, cholesterol/ high-density lipoprotein (HDL) ratio, glycosylated haemoglobin (HbA1c), material deprivation, ethnicity, smoking, diabetes duration, type of diabetes, atrial fibrillation, cardiovascular disease, chronic renal disease, and family history of premature coronary heart disease.

**Methods:**

We used Cox proportional hazards models to derive separate risk equations in men and women for evaluation at 10 years. Measures of calibration, discrimination, and sensitivity were determined in 2 external validation cohorts.

**Results:**

We identified 25 480 cases of heart failure in the derivation cohort, 8189 in the QResearch validation cohort, and 11 311 in the CPRD cohort. The equations included: age, BMI, systolic blood pressure, cholesterol/HDL ratio, HbA1c, material deprivation, ethnicity, smoking, duration and type of diabetes, atrial fibrillation, cardiovascular disease, and chronic renal disease. The equations had good performance in CPRD for women (R^2^ of 41.2%; D statistic 1.71; and receiver operating characteristic curve (ROC) statistic 0.78) and men (38.7%, 1.63; and 0.77 respectively).

**Conclusions:**

We have developed and externally validated risk prediction equations to quantify absolute risk of heart failure in men and women with diabetes. These can be used to identify patients at high risk of heart failure for prevention or assessment of the disease.

**Strengths and limitations of this study**

- The algorithms provide valid measures of absolute risk of heart failure in patients with type 1 or type 2 diabetes, as shown by the performance in a separate validation cohort.
- Key strengths include size, duration of follow-up, representativeness, and lack of selection, recall and respondent bias.
- The study has good face validity since it has been conducted in the setting where the majority of patients in the UK are assessed, medically treated, and followed up.
- The study used linked hospital and mortality data records and is, therefore, likely to have picked up the majority of heart failure diagnoses.
- The algorithms do not include some biomarkers for heart failure, such as B-type natriuretic peptide, as these are not routinely recorded in electronic health records.

## Introduction

Cardiovascular disease is a major cause of morbidity and decreased life expectancy in patients with diabetes. Clinical guidelines recommend cardiovascular risk assessment in patients with diabetes^1^ ^2^ to assist with prevention of heart failure. Cardiovascular risk assessment tools, including Framingham^3^ and QRISK2,^4^ are used both for communicating risk to patients with diabetes and to guide public health decisions.^1^ ^2^ Considerable attention—in clinical trials, guidelines and observational studies^5^—is focused on predicting and preventing cardiovascular disease (particularly coronary heart disease and stroke), reflecting perceptions that it is the main disease burden for diabetes.^6^ ^7^

Less emphasis has been given to the development of risk assessment methods to predict heart failure among people with diabetes although it is recognised to be a substantial cause of morbidity and mortality requiring different investigations and treatments.^6^ ^8^ ^9^ It is associated with significant morbidity and healthcare costs.^8^ Heart failure has also been associated as an adverse event with selected antihyperglycaemic agents used to treat diabetes.^10^ Once heart failure is present in individuals with diabetes mellitus, there is a 10-fold increase in mortality and a 5-year survival of only 12.5%, a prognosis worse than metastatic breast cancer.^11^

We have developed and validated various risk prediction equations, including QRISK2^4^ for cardiovascular disease, and QStroke^12^ for stroke, designed to quantify an individual's absolute risk of these clinically important outcomes based on risk factors recorded in linked electronic health records. These risk equations are used internationally in patients with and without diabetes to assist in decisions regarding further investigation, treatment or referral. While there have been several risk scores to predict incident heart failure applicable to patients with diabetes,^9^ ^13^ ^14^ these are not based on contemporaneous ethnically diverse populations, only cover limited age ranges (eg, 45–64 years) or time periods,^13^ include very few patients with diabetes,^13^ or are limited only to small numbers of patients with hospitalisations for heart failure.^9^ None are currently in widespread use for everyday clinical practice in the UK.

The objective of the present study is to derive and externally validate new risk prediction equations to quantify 10-year absolute risk of heart failure in patients with diabetes by using linked electronic health records. The purpose of the equations is to better quantify absolute risk of heart failure in order to prompt (1) closer management of modifiable risk factors, (2) earlier diagnosis of heart failure in ‘at risk’ patients, and (3) to provide better information for patients and doctors to inform treatment decisions about antihyperglycaemic agents which might also inadvertently increase risk of heart failure.

## Methods

### Study design and data source

We undertook a cohort study to derive and validate the risk equations in a large population of primary care patients using the large QResearch primary care database (V.39, http://www.qresearch.org). QResearch is a continually updated patient-level pseudonymised database with event-level data extending back to 1989. QResearch includes clinical and demographic data from over 1000 general practices covering a population of >20 million patients, collected in the course of routine healthcare by general practitioners and associated staff. The primary care data includes demographic information, diagnoses, prescriptions, referrals, laboratory results and clinical values. Diagnoses are recorded using the Read code classification. QResearch has been used for a wide range of clinical research including the development and validation of risk prediction models.^12^ ^15^ The primary care data is linked at individual patient level to Hospital Episode Statistics (HES), and mortality records from the Office for National Statistics (ONS). HES provides details of all National Health Service (NHS) inpatient admissions since 1997, including primary and secondary causes coded using the International Classification of Diseases, 10th Revision (ICD-10) classifications. ONS provides details of all deaths in England with primary and underlying causes, also coded using the ICD-10 classification. Patient records are linked using a project-specific pseudonymised NHS number which is valid and complete for 99.8% of primary care patients, 99.9% for ONS mortality records, and 98% for hospital admissions records.

We included all QResearch practices in England who had been using their Egton Medical Information Systems (EMIS) computer system for at least a year. We randomly allocated three quarters of these practices to the derivation data set and the remaining quarter to a validation data set. In both data sets, we identified open cohorts of patients aged 25–84 years registered with eligible practices between 1 January 1998 and 31 July 2014. We then selected patients with diabetes if they had a Read code for diabetes or more than one prescription for insulin or oral hypoglycaemics. We classified patients as having type 1 diabetes if they had been diagnosed under the age of 35 years and prescribed insulin.^16^ We excluded patients without a postcode-related deprivation score and patients with an existing diagnosis of heart failure. We determined an entry date to the cohort for each patient, which was the latest of the following dates: 25th birthday, date of registration with the practice plus 1 year, date on which the practice computer system was installed plus 1 year, date of diagnosis of diabetes, and the beginning of the study period (1 January 1998). Patients were censored at the earliest date of the diagnosis of heart failure, death, de-registration with the practice, the last upload of computerised data, or the study end date (1 August 2014).

We undertook a second external validation using general practices in England contributing to the Clinical Research Practice Datalink (CPRD). CPRD is a similar database to QResearch except that it is derived from practices using a different clinical computer system. We used the subset of 357 CPRD practices which were linked to ONS mortality and hospital admissions. We used the same definitions for selecting a validation cohort as done for QResearch except that the study end date was 1 August 2012, the latest date for which linked data were available.

### Outcome

We classified patients as having incident heart failure if there was a record of the relevant diagnosis either in their primary care record, their linked hospital record, or mortality record. We used Read codes to identify recorded clinical diagnoses of heart failure from the primary care record (G58%,G5yy9,G5yyA,662f,662g,662h,662i). We used ICD-10 clinical codes (I110, I130, I42, I50) to identify incident cases of heart failure from hospital and mortality records. We used the earliest recorded date of heart failure on any of the three data sources as the index date for the diagnosis of heart failure.

### Predictor variables

We examined the following predictor variables based on established risk factors for cardiovascular disease and heart failure.^14^ ^15^ ^17^ ^18^ We focused on variables likely to be recorded as coded data in the patient's electronic record. For continuous variables, we used the closest values recorded prior to the baseline date or within 6 months after the cohort entry date. All other predictor variables were based on the latest information recorded in the primary care record before entry to the cohort. We did not include natriuretic peptide levels since this is not routinely measured or recorded in electronic health records.

- Age at entry to cohort (continuous).
- Type of diabetes: type 1 or type 2.
- Number of years since diagnosis of diabetes (<1 year; 1–3; 4–6; 7–10 and ≥11 years).
- Smoking status (non-smoker; ex-smoker; light smoker (1–9 cigarettes/day); moderate smoker (10–19 cigarettes/day); heavy smoker (20+ cigarettes/day).
- Ethnic group (Caucasian/not recorded, Indian, Pakistani, Bangladeshi, Other Asian, black Caribbean, black African, Chinese, Other).^15^
- Townsend deprivation score (continuous)^15^ where a higher score indicates greater levels of material deprivation.
- Family history of premature coronary heart disease (in a first-degree relative aged less than 60 years).^15^
- Chronic renal disease^19^ (using Read codes for chronic kidney disease (CKD) 4 or CKD5; nephrotic syndrome; chronic glomerulonephritis; chronic pyelonephritis; end-stage renal failure; renal transplant; dialysis).
- Cardiovascular disease (coronary artery disease or cerebrovascular disease).^19^
- Atrial fibrillation.^15^
- Treated hypertension.^15^
- Rheumatoid arthritis.^15^
- Body mass index (BMI) kg/m^2^(continuous).^19^
- Cholesterol/high-density lipoprotein (HDL) level (continuous).^15^
- Systolic blood pressure (continuous).^15^
- Glycosylated haemoglobin (HbA1c) mmol/mol (continuous).^19^ ^20^

### Derivation of the models

We developed the risk prediction equations in the derivation cohort using established methods.^15^ ^21^ We used fractional polynomials^22^ based on a complete case analysis to model non-linear risk relationships with continuous variables (age, BMI, systolic blood pressure, cholesterol/HDL ratio, HbA1c), where appropriate. We used multiple imputation to replace missing values for continuous values (BMI, systolic blood pressure, cholesterol/HDL ratio, HbA1c) and smoking status, and used these values in our main analyses.^23^ ^24^ We carried out 10 imputations and included all of the candidate predictor variables in the imputation model along with the outcome variable and logarithm of person time. We used Cox's proportional hazards models to estimate the coefficients for each risk factor for men and women separately. We used Rubin's^25^ rules to combine the regression coefficients across the imputed data sets. We fitted full models initially then retained variables if these had a HR of <0.80 or >1.20 (for binary variables) and were statistically significant at the 0.05 level. We examined interactions between predictor variables and age, and included these where they were significant, plausible, and improved model fit. We used regression coefficients for each variable from the final model as weights which we combined with the baseline survivor function evaluated up to 15 years to derive risk equations for over a period of 15 years of follow-up.^26^ This enabled us to derive risk estimates for each year of follow-up, with a specific focus on 10-year risk estimates. We estimated the baseline survivor function based on zero values of centred continuous variables, with all binary predictor values set to zero. Model fit was assessed by measuring the Akaike information criterion (AIC) and Bayesian information criterion (BIC) values for each imputed set of data.

### Validation of the models

We used multiple imputation in the two validation cohorts to replace missing values for continuous variables and smoking status. We carried out 10 imputations and included the candidate predictor variables in the imputation model along with the outcome variable and the logarithm of person time. We applied the risk equations for men and women obtained from the derivation cohort to the validation cohorts, and calculated measures of discrimination. We calculated R^2^ values (explained variation in time to diagnosis of heart failure^27^), D statistics^28^ (a measure of discrimination where higher values indicate better discrimination) and the area under the receiver operating characteristic curve (ROC) statistic at 10 years, and combined the model performance measures across data sets using Rubin's rules. We assessed calibration using one imputed data set (comparing the mean predicted risks at 10 years with the observed risk by tenth of predicted risk). The observed risks were obtained using Kaplan-Meier estimates evaluated at 10 years. We applied the equations to the validation cohorts to define thresholds for the 10% of patients at highest estimated risk of heart failure at 10 years.

We used all the available data on each database to maximise the power and also generalisability of the results. We used STATA (V.13.1) for all analyses. The TRIPOD statement was adhered to in reporting.^29^

## Results

### Overall study population

Overall, 1017 QResearch practices in England met our practice inclusion criteria, of which 763 were randomly assigned to the derivation data set with the remaining 254 practices assigned to the validation cohort. We identified 455 551 patients aged 25–84 years with diabetes in the derivation cohort. We excluded 976 patients (0.21%) without a recorded Townsend deprivation score and 16 769 (3.68%) with a diagnosis of heart failure at baseline, leaving 437 806 for analysis.

We identified 142 718 patients aged 25–84 years with diabetes in the QResearch validation cohort. We excluded 299 patients (0.21%) without a recorded Townsend deprivation score and 5391 (3.78%) with a diagnosis of heart failure at baseline, leaving 137 028 for validation.

We identified 206 050 patients aged 25–84 years with diabetes in the CPRD validation cohort from the 357 practices with linked hospital and mortality data. We excluded 8145 patients (3.95%) with a diagnosis of heart failure at baseline, leaving 197 905 for validation.

### Baseline characteristics

Table 1 shows baseline characteristics of 437 806 patients with diabetes in the derivation cohort free from heart failure at study entry. Of these, 412 556 (94.2%) had type 2 diabetes and 25 250 (5.8%) had type 1 diabetes, 35.6% had been treated for hypertension, 17.4% had cardiovascular disease, 1.0% had chronic renal disease, and 3.2% had atrial fibrillation. Just over half (54.2%) had been diagnosed with diabetes for less than a year at cohort entry, 17.0% had been diagnosed for 1–3 years, 9.4% for 4–6 years, 7.8% for 7–10 years, and 11.5% for 11 or more years before cohort entry. Smoking was recorded in 95.3% of patients, ethnicity in 75.7%, BMI in 90.8%, systolic blood pressure in 97.0%, HbA1c in 70.9% and cholesterol/HDL in 53.8%. Complete data for all clinical values, smoking, and ethnicity were available for 182 477 (41.7%) of the derivation cohort.

**Table 1:** Baseline characteristics of patients with diabetes aged 25–84 years and without heart failure at baseline in the QResearch derivation cohort and both validation cohorts

	QResearch derivation cohort (%)	QResearch validation cohort (%)	CPRD validation cohort (%)
Total number of patients without heart failure at baseline	437 806 (100)	137 028 (100)	197 905 (100)
Women	192 896 (44.1)	60 152 (43.9)	86 776 (43.8)
Men	244 910 (55.9)	76 876 (56.1)	111 129 (56.2)
Type 1 diabetes	25 250 (5.8)	7803 (5.7)	11 021 (5.6)
Type 2 diabetes	412 556 (94.2)	129 225 (94.3)	186 884 (94.4)
Diet control*	156 272 (35.7)	50 580 (36.9)	60 174 (32.2)
Oral hypoglycaemics only*	228 235 (52.1)	70 301 (51.3)	113 007 (60.5)
Oral hypoglycaemics+insulin*	399 (0.1)	105 (0.1)	197 (0.1)
Insulin only*	27 650 (6.3)	8239 (6.0)	13 506 (7.2)
*Years since diagnosis of diabetes*			
<1	237 341 (54.2)	76 453 (55.8)	107 944 (54.5)
1–3	74 638 (17.0)	22 023 (16.1)	33 016 (16.7)
4–6	41 042 (9.4)	12 228 (8.9)	18 027 (9.1)
7–10	34 237 (7.8)	10 616 (7.7)	15 696 (7.9)
≥11	50 548 (11.5)	15 708 (11.5)	23 222 (11.7)
Mean age (SD)	60.0 (13.7)	60.4 (13.7)	61.0 (13.3)
Mean Townsend deprivation score (SD)†	0.6 (3.4)	0.2 (3.3)	−0.2 (3.3)
*Ethnicity*			
Ethnicity recorded	331 358 (75.7)	102 660 (74.9)	89 073 (45.0)
Ethnicity not recorded	106 448 (24.3)	34 368 (25.1)	110 519 (55.0)
Caucasian/not recorded‡	363 702 (83.1)	118 727 (86.6)	184 564 (93.3)
Indian	15 534 (3.5)	4431 (3.2)	3468 (1.8)
Pakistani	10 513 (2.4)	1879 (1.4)	1552 (0.8)
Bangladeshi	12 444 (2.8)	1951 (1.4)	580 (0.3)
Other Asian	7072 (1.6)	2374 (1.7)	1920 (1.0)
Black Caribbean	9975 (2.3)	2797 (2.0)	1643 (0.8)
Black African	8357 (1.9)	2074 (1.5)	1718 (0.9)
Chinese	1396 (0.3)	409 (0.3)	305 (0.2)
Other	8813 (2.0)	2386 (1.7)	2155 (1.1)
*Smoking status*			
Smoking status recorded	417 286 (95.3)	130 892 (95.5)	195 712 (98.9)
Non-smoker	219 640 (50.2)	67 736 (49.4)	81 393 (41.1)
Ex-smoker	118 560 (27.1)	38 750 (28.3)	39 936 (20.2)
Light smoker (1–9 cigarettes/day)	44 843 (10.2)	13 744 (10.0)	12 200 (6.2)
Moderate smoker (10–19 cigarettes/day)	17 943 (4.1)	5575 (4.1)	21 790 (11.0)
Heavy smoker (20+ cigarettes/day)	16 300 (3.7)	5087 (3.7)	16 801 (8.5)
Smoker amount not recorded	0 (0.0)	0 (0.0)	23 592 (11.9)
*Family history and comorbidity*			
Family history of premature CHD	61 914 (14.1)	20 436 (14.9)	11 014 (5.6)
Rheumatoid arthritis	11 303 (2.6)	3440 (2.5)	2984 (1.5)
Atrial fibrillation	13 953 (3.2)	4593 (3.4)	6676 (3.4)
Cardiovascular disease	76 377 (17.4)	24 299 (17.7)	37 800 (19.1)
Treated hypertension	155 869 (35.6)	48 580 (35.5)	60 311 (30.5)
Chronic renal disease	4302 (1.0)	1352 (1.0)	1740 (0.9)
*Clinical values*			
HbA1c recorded	310 356 (70.9)	96 061 (70.1)	114 920 (58.1)
Mean HbA1c mmol/mol (SD)	62.4 (21.5)	62.2 (21.5)	61.9 (21.7)
BMI recorded	397 409 (90.8)	124 129 (90.6)	183 301 (92.6)
Mean BMI, kg/m^2^ (SD)	30.3 (5.8)	30.4 (5.8)	30.2 (5.8)
Cholesterol/HDL ratio recorded	235 540 (53.8)	74 671 (54.5)	79 193 (40.0)
Mean cholesterol/HDL ratio (SD)	4.3 (1.4)	4.3 (1.4)	4.4 (1.6)
Systolic blood pressure recorded	424 623 (97.0)	133 157 (97.2)	194 340 (98.2)
Mean systolic blood pressure mm Hg (SD)	138.9 (19.2)	139.3 (19.2)	140.7 (19.7)

Values are numbers (percentages) unless stated otherwise.

*Per cent given are of all patients in total cohort.

†The Townsend score ranges between −8 and +11; increasing levels of Townsend scores indicate increasing levels of material deprivation.

‡Includes patients who were either recorded as Caucasian or those without any ethnicity recorded.

BMI, body mass index; CHD, coronary heart disease; HbA1c, glycosylated haemoglobin; HDL, high-density lipoprotein.

Online supplementary table 1 shows the baseline characteristics of the derivation cohort in men and women separately. Online supplementary table 2 shows the distribution of patients throughout the 10 geographical regions of England. Baseline characteristics for patients in the QResearch validation cohort were similar to corresponding values in the derivation cohort with 41.7% of patients having complete data for all clinical values, smoking, and ethnicity. Baseline characteristics of the CPRD validation cohort were also similar except that the recording of ethnicity (45.0%) and HbA1c (58.1%) was substantially lower in CPRD. In total, 19.4% of CPRD patients had complete data for all candidate predictor variables.

### Primary outcome of heart failure

Table 2 shows the number of incident cases of heart failure during follow-up and the directly age standardised incidence rates in each cohort. There were 25 480 cases of heart failure in the QResearch derivation cohort, 8189 in the QResearch validation cohort, and 11 311 in the CPRD validation cohort.

**Table 2:** Numbers of incident cases of HF during follow-up and age standardised incidence rates per 1000 person years in men and women with diabetes aged 25–84 years in the derivation cohort and validation cohorts

	QResearch derivation cohort		QResearch validation cohort		CPRD validation cohort	
	Total HF cases	Rate per 1000 person years (95% CI)	Total HF cases	Rate per 1000 person years (95% CI)	Total HF cases	Rate per 1000 person years (95% CI)
Women	10 710	9.09 (8.92 to 9.26)	3395	8.99 (8.68 to 9.29)	4827	9.05 (8.79 to 9.31)
Men	14 770	11.66 (11.47 to 11.85)	4794	11.79 (11.46 to 12.12)	6484	11.44 (11.16 to 11.72)

Rates were directly age standardised to the overall age distribution of patients aged 25–84 years within the QResearch derivation cohort in 5-year age bands.

HF, heart failure.

### Predictor variables

**Figure 1:**
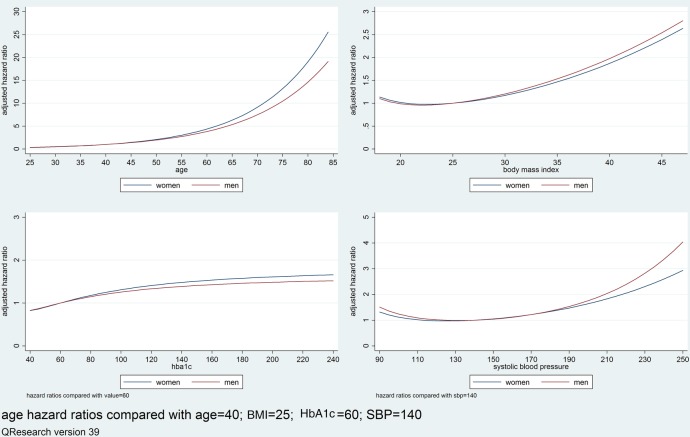
Graphs of the adjusted HRs for the fractional polynomial (FP) terms for age, body mass index (BMI), glycosylated haemoglobin (HbA1c) and systolic blood pressure (SBP) in the derivation cohort.

Table 3 shows adjusted HRs for variables in the final models for men and women in the derivation cohort which included: age, BMI, systolic blood pressure, cholesterol/HDL, HbA1c, deprivation, duration and type of diabetes, smoking status, ethnicity, atrial fibrillation, cardiovascular disease and chronic renal disease (the univariate associations are shown in online supplementary table 3). Increasing duration of diabetes was associated with increased heart failure risk despite adjustment for current age and other risk factors. Non-Caucasian ethnic groups tended to have a lower risk of heart failure compared with those whose ethnic group was either Caucasian or not recorded. There was a ‘dose–response’ relationship for smoking with heavy smokers having the highest risk of heart failure. In women, type 1 diabetes had a 38% higher risk of heart failure compared with type 2; atrial fibrillation: 143% higher risk; cardiovascular disease: 96% increased risk; and chronic renal disease: 64% increased risk. The pattern was similar for men. Figure 1 shows the adjusted HRs using fractional polynomial terms for continuous variables. Increasing age, BMI, systolic blood pressure, and HbA1c were all associated with increasing HRs of heart failure.

**Table 3:** Adjusted HRs with 95% CIs for heart failure in men and women in the derivation cohort

	Adjusted HR (95% CI)	
	Women*	Men†
Age‡	1.076 (1.074 to 1.079)	1.069 (1.067 to 1.071)
Cholesterol/HDL ratio‡	1.04 (1.01 to 1.06)	1.03 (1.01 to 1.04)
Townsend deprivation score‡,§	1.25 (1.21 to 1.28)	1.17 (1.14 to 1.20)
Duration of diabetes at baseline (years)		
<1	1	1
1–3	1.45 (1.38 to 1.53)	1.29 (1.23 to 1.35)
4–6	1.53 (1.44 to 1.63)	1.47 (1.39 to 1.55)
7–10	1.72 (1.62 to 1.84)	1.49 (1.41 to 1.58)
≥11	1.94 (1.83 to 2.07)	1.72 (1.63 to 1.81)
Smoking status		
Non-smoker	1	1
Ex-smoker	1.10 (1.05 to 1.15)	1.03 (0.99 to 1.07)
Light smoker (1–9 cigarettes/day)	1.33 (1.23 to 1.43)	1.34 (1.27 to 1.41)
Moderate smoker (10–19 cigarettes/day)	1.52 (1.38 to 1.69)	1.35 (1.23 to 1.48)
Heavy smoker (20+ cigarettes/day)	1.74 (1.55 to 1.95)	1.51 (1.38 to 1.66)
Ethnicity		
Caucasian/not recorded	1	1
Indian	1.01 (0.89 to 1.16)	0.94 (0.84 to 1.05)
Pakistani	1.09 (0.92 to 1.29)	0.92 (0.80 to 1.06)
Bangladeshi	0.94 (0.78 to 1.13)	1.14 (1.00 to 1.30)
Other Asian	0.96 (0.76 to 1.22)	0.77 (0.63 to 0.95)
Black Caribbean	0.73 (0.64 to 0.83)	0.77 (0.68 to 0.88)
Black African	0.91 (0.72 to 1.16)	0.68 (0.53 to 0.87)
Chinese	0.78 (0.47 to 1.29)	0.41 (0.24 to 0.70)
Other ethnic group	0.82 (0.68 to 0.99)	0.74 (0.62 to 0.87)
Comorbidity		
Type 1 diabetes¶	1.38 (1.20 to 1.58)	1.19 (1.06 to 1.34)
Atrial fibrillation**	2.43 (2.27 to 2.60)	2.07 (1.95 to 2.19)
Cardiovascular disease**	1.96 (1.88 to 2.04)	2.19 (2.12 to 2.27)
Chronic renal disease**	1.64 (1.40 to 1.91)	1.77 (1.57 to 2.01)

For FP terms see footnotes and figure 1.

*Heart failure model in women included body mass index (2 FP terms −1 −.5), systolic blood pressure (2 FP terms −.5 0), hba1c (2 FP terms −2 −2).

†Heart failure model in men included body mass index (2 FP terms −2 0), systolic blood pressure (2 FP terms 0 .5), hba1c (2 FP terms −2 −2).

‡Adjusted HR is per unit increase.

§The Townsend deprivation score ranges between −7 (most affluent) and +11 (most deprived).

¶Adjusted HR compared with type 2 diabetes.

**Adjusted HR compared with patients without the condition at baseline.

FP, fractional polynomial; HbA1c, glycosylated haemoglobin; HDL, high-density lipoprotein.

The full equation for the model is published as opensource software at http://qdiabetes.org/heart-failure/index.php.

### Validation

#### Discrimination

Table 4 shows the performance of each equation in each validation cohort for women and men separately. In the CPRD validation cohort, in women the algorithm explained 41.2% of the variation in time to diagnosis of heart failure (R^2^), and discrimination was good with a D statistic of 1.71 and ROC value of 0.78. Corresponding values for men were 38.72%, 1.63 and 0.77, respectively. The results of the validation in the QResearch validation cohort were very similar.

**Table 4:** Performance of the equations in men and women in QResearch validation cohort and CPRD validation cohort

Statistic	QResearch validation cohort Mean (95% CI)	CPRD validation cohort Mean (95% CI)
Women		
D statistic	1.66 (1.60 to 1.72)	1.71 (1.66 to 1.76)
R^2^	39.77 (38.07 to 41.46)	41.15 (39.72 to 42.58)
ROC	0.770 (0.762 to 0.778)	0.783 (0.776 to 0.789)
Men		
D statistic	1.67 (1.62 to 1.72)	1.63 (1.58 to 1.67)
R^2^	40.04 (38.54 to 41.53)	38.72 (37.44 to 40.00)
ROC	0.764 (0.757 to 0.771)	0.769 (0.763 to 0.775)

Discrimination is the ability of the risk prediction model to differentiate between patients who experience an admission event during the study and those who do not. This measure is quantified by calculating the area under the ROC statistic; where a value of 0.5 represents chance and 1 represents perfect discrimination.

The D statistic is also a measure of discrimination which is specific to censored survival data. As with the ROC, higher values indicate better discrimination. R^2^ measures explained variation and higher values indicate more variation is explained.

ROC, receiver operating characteristic curve.

#### Calibration

**Figure 2:**
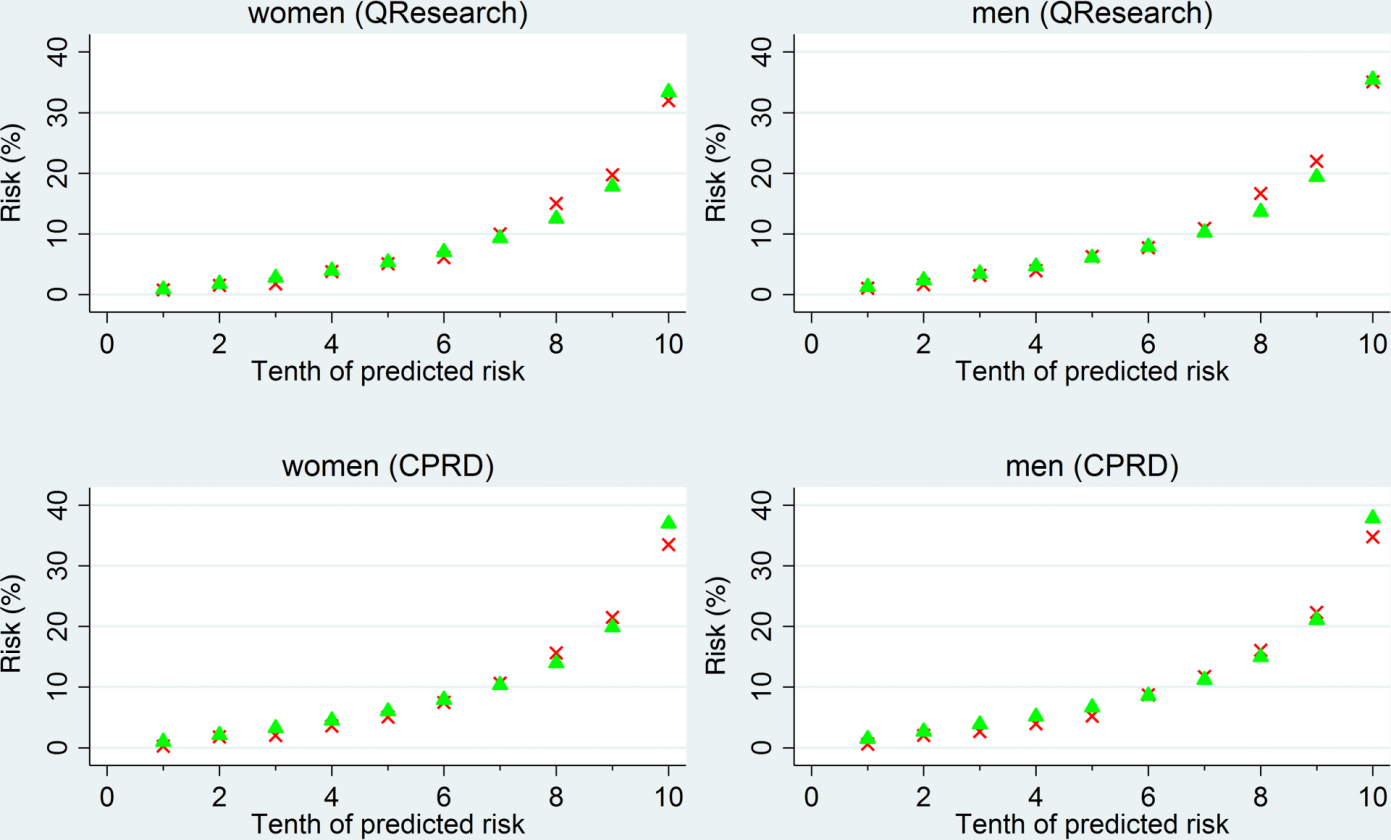
The mean predicted risks and observed risks of heart failure at 10 years, by tenth of predicted risk, applying the equations to all men and women in the QResearch and Clinical Research Practice Datalink (CPRD) validation cohorts. Crosses denote 10-year observed risk; triangles denote 10-year predicted risk.

Figure 2 shows the mean predicted risks and observed risks of heart failure at 10 years—by tenth of predicted risk—applying the equations to all men and women in the QResearch and CPRD validation cohorts. There was close correspondence between the mean predicted risks and the observed risks within each model tenth in women and men indicating that the equations were well calibrated across both validation cohorts.

#### Performance at threshold for the 10% at highest risk

The sensitivity, specificity, and observed risk for the 10% of women at highest predicted risk of heart failure in the QResearch validation cohort (ie, cut-off for 10-year risk of heart failure ≥21.8%) were 31.5%, 91.1%, and 32.0% respectively. The corresponding figures for men (using a 10-year heart failure risk threshold of ≥ 23.9%) were 30.6%, 91.2%, and 35.1%, respectively. The corresponding results for women on CPRD were 33.5%, 91.3% and 33.5%, respectively, and for men, 31.9%, 91.2% and 34.7%, respectively.

**Figure 3:**
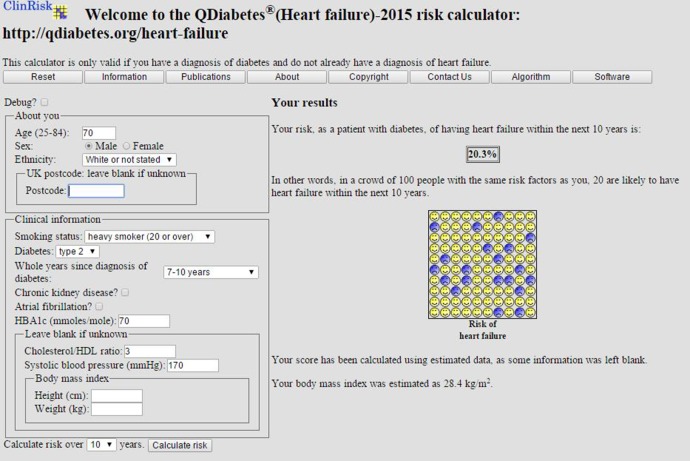
Web calculator applied to an example patient.

Figure 3 shows a clinical example of the implementation of the equation as a web calculator. For international users the postcode field can be left blank.

## Discussion

### Key findings

We have developed and externally validated risk prediction equations to quantify absolute 10-year risk of heart failure in men and women with either type 1 or type 2 diabetes. To the best of our knowledge, this is the first equation to predict 10-year risk of heart failure in men and women with diabetes based on a very large representative ethnically diverse contemporaneous population. The equations include 13 variables: age, BMI, systolic blood pressure, cholesterol/HDL ratio, HbA1c, material deprivation, ethnicity, smoking, duration and type of diabetes, atrial fibrillation, cardiovascular disease and chronic renal disease. The equations were well calibrated and had good discrimination with ROC values exceeding 0.75 in both validation cohorts. The purpose of the equation is to better quantify absolute risk of heart failure in order to prompt (1) closer management of modifiable risk factors, such as smoking status, systolic blood pressure, BMI and cholesterol, using appropriate interventions for lifestyle or with medication; (2) earlier diagnosis and treatment of heart failure in ‘at risk’ patients and (3) to provide better information for patients and doctors to inform the treatment decisions about antihyperglycaemic agents which might also inadvertently increase risk of heart failure.

### Comparisons with the literature

We included established risk factors in our equation and demonstrated HRs similar in both magnitude and direction to those reported for heart failure elsewhere,^19^ ^14^ which increases the clinical face validity of the equations. Cardiovascular disease was associated with a twofold increased risk of heart failure, as in the study of 6496 patients with diabetes by Nichols *et al*.^19^ Chronic renal disease, increasing BMI, increasing systolic blood pressure^29^ and HbA1c level,^20^ and a longer duration of diabetes were associated with higher risk of heart failure.^19^ Compared with the Atherosclerosis Risk in Communities (ARIC) study based on 15 792 US patients aged 45–64 years with 1487 heart failure events^14^ and the study of 7067 Hong Kong patients with 274 heart failure events,^9^ we have included a much larger, more contemporaneous ethnically diverse population of patients with either type 1 or type 2 diabetes and spanning a wider age range. We have included additional predictors such as atrial fibrillation, chronic renal disease, and HbA1c known to be associated with risk of heart failure. Our ROC values were similar to those from ARIC,^14^ lower than those reported from a study of patients from Hong Kong^9^ but higher than that reported for the published Framingham heart failure score.^14^

### Methodological considerations

The methods used to derive and validate these models are the same as for a range of other clinical risk prediction tools derived from the QResearch database.^12^ ^15^ ^30–32^ The strengths and limitations of the approach have been discussed in detail,^15^ ^21^ including information on multiple imputation of missing data. In summary, key strengths of these models include size, duration of follow-up, representativeness, and lack of selection, recall and respondent bias. UK general practices have good levels of accuracy and completeness in recording clinical diagnoses and prescribed medications.^33^ Our database has linked hospital and mortality records for nearly all patients and is, therefore, likely to have picked up the majority of cases of diagnosed heart failure thereby minimising ascertainment bias. We undertook two validations, one using a separate set of practices and patients contributing to QResearch, and the other using a fully external set of practices contributing to CPRD.

The results of both validations were extremely similar, which is consistent with previous validation studies showing comparable performance using different populations.^21^ ^34^ While we have derived and validated the score using UK data sets, the score can be used internationally by leaving the postcode deprivation field blank (in which case a mean value would be substituted) or by creating an equivalent measure to the Townsend score for local use. Best practice would be to undertake a validation using a relevant population before local use to ensure good calibration in the applicable population.

Limitations of our study include the lack of formal adjudication of diagnoses, and potential for bias due to missing data. However, we think our ascertainment of diagnosed heart failure is likely to be high given the combination of the three linked data sources although we may have missed clinically silent heart failure. We did not include natriuretic peptide levels since this is not routinely measured or recorded in electronic health records. We have not provided definite comment on what threshold of absolute risk should be used to define a “high risk” group as that would require (1) the balance of risks and benefits for individuals and (2) cost-effectiveness analyses which are outside the scope of this study.

## Conclusion

We have developed and validated a new risk prediction equation to quantify the absolute risk of heart failure in patients with type 1 or type 2 diabetes. It can be used to identify patients with diabetes at high risk of heart failure for further assessment and proactive treatment.
